# Chemical Composition and Deposition Fluxes of Water-Soluble Inorganic Ions on Dry and Wet Deposition Samples in Wuhan, China

**DOI:** 10.3390/ijerph16010132

**Published:** 2019-01-06

**Authors:** Jun Qin, Yassin Mbululo, Muyi Yang, Zhengxuan Yuan, Fatuma Nyihirani, Xiang Zheng

**Affiliations:** 1 School of Environmental Studies, China University of Geosciences, 388 Lu Mo Road, Wuhan 430074, China; qinjun@cug.edu.cn (J.Q.); 15926426505@163.com (M.Y.); 18602758378@163.com (Z.Y.); fnyihirani@mzumbe.ac.tz (F.N.); zhengxiang@cug.edu.cn (X.Z.); 2 Department of Geography and Environmental Studies, Solomon Mahlangu College of Science and Education, Sokoine University of Agriculture, P.O. Box 3038, Morogoro, Tanzania; 3 Centre for Environment, Poverty and Sustainable Development, Mzumbe University, P.O. Box 83, Morogoro, Tanzania

**Keywords:** air quality, dry deposition, wet deposition, deposition flux, water-soluble inorganic ions

## Abstract

Measurement of PM_2.5_ concentration, dry and wet deposition of water-soluble inorganic ions (WSII) and their deposition flux was carried out. During sampling, a total number of 31 samples of PM_2.5_, five wet deposition samples and seven dry deposition samples were collected. The analyses results showed that the average concentration of PM_2.5_ was 122.95 µg/m^3^ whilst that of WSII was 51.63 µg/m^3^, equivalent to 42% of the total mass of PM_2.5_. The correlation coefficients between WSII in samples of PM_2.5_ was significant (r = 0.50 and *p*-value of 0.0019). Ions of ${\;\mathrm{SO}}_{4}^{2-}$, ${\mathrm{NO}}_{3}^{-}$, ${\mathrm{Cl}}^{-}$, and ${\;\mathrm{NH}}_{4}^{+}$ were dominant in the entire samples (PM_2.5_, dry and wet depositions), nevertheless, the average concentration of both ${\mathrm{SO}}_{4}^{2-}$ and ${\mathrm{Cl}}^{-}$ were below the China environmental quality standard for surface water. The ratio of dominant anions in wet deposition (${\mathrm{SO}}_{4}^{2-}$/${\mathrm{NO}}_{3}^{-}$) was 1.59, whilst that for dry deposition (${\mathrm{SO}}_{4}^{2-}$/${\mathrm{Cl}}^{-}$) was 1.4, indicating that acidity was mainly derived from sulphate. In the case of dominant cations, the dry and wet deposition ratios (${\mathrm{Ca}}^{2+}$/${\mathrm{NH}}_{4}^{+}$) were 1.36 and 1.37, respectively, suggesting the alkaline substances were mainly dominated by calcium salts. Days with higher recorded concentrations of PM_2.5_ were accompanied by dry and warm boundary layer structure, weak low-level wind and strong inversion layer.

## 1. Introduction

Dry and wet deposition are basically two main processes for removal of water-soluble inorganic ions (WSII) from the atmosphere. These WSII encompass significant portions of PM_2.5_ (particulate matter with aerodynamics diameter of equal to or less than 2.5 microns) and precipitation samples, thus, they can directly impact air quality, climate and the ecosystem at large [1,2]. Dry deposition process includes Brownian motion of particles, settling by gravity and impact of wind, while the wet deposition process depends on precipitation scavenging (i.e., rain, snow, hail, clouds, fog etc.) [3,4,5,6]. Even though, precipitation scavenging is the prime mechanism for pollutants (gaseous and particulate) removal from the atmosphere [7], study by Gambaro [8] show that dry deposition to be more constant with time. Usually, the maximum dry deposition flux are observed when the wet deposition flux reach its minimum value. Elsewhere in the world, significant work has been done to study the sources [9], chemical composition [10] and dry and wet depositions [3,5,6,8,11,12] of these WSII as compared to China. Nevertheless, study by Inomata et al. [11] reveal non-existence of a generally accepted technique for dry deposition sampling because each technique has its own degree of uncertainty in estimating the deposition.

China as one of the developing countries with the fast growing economy is facing a serious air pollution problem in most of its cities. The status of air quality is always worse during winter due to PM_2.5_ pollution, and during summer due to ozone pollution. As a mitigation measure for the persistent pollution, the Chinese government introduced the National Ambient Air Quality Standard (NAAQS) in 2012, which for the first time included PM_2.5_ as a monitored indicator in Air Quality Index (AQI). It is worth noting that, apart from other constituent species which make PM_2.5_, WSII in China account for more than one third of the total mass composition of PM_2.5_ [13,14]. A study by Huang et al. [15] went far by associating a small difference in mass concentration of WSII in different localities to geographical and meteorological conditions and energy structure. So far, a number of studies have been done in China with regard to WSII [2,13,14,15,16,17,18]. For instance, study by Li et al. [2] in Huangshan, Southeast China found WSII concentration were decreasing with altitude. The dominant ions were ${\mathrm{SO}}_{4}^{2-}$, ${\mathrm{NO}}_{3}^{-}$ and ${\mathrm{NH}}_{4}^{+}$ at both higher and lower elevation occupying 71% and 78% of total WSII, respectively. Diurnal variation of dominant ions in Beijing show the peak time of ${\mathrm{NO}}_{3}^{-}$ was during morning and night time while ${\mathrm{SO}}_{4}^{2-}$ and ${\mathrm{NH}}_{4}^{+}$ peaked at noon [13]. The observed peak values of ${\mathrm{NH}}_{4}^{+}$ at noon were associated with emission from vehicles. A study by Yang et al. [16] revealed the pollutants of nitrogen origin were higher in Guangzhou than Beijing, Chongqing and Shanghai based on the calculated equivalent ratios of ${\mathrm{NO}}_{3}^{-}$/${\mathrm{SO}}_{4}^{2-}$. This means that, mobile source pollution accounted more into PM_2.5_ concentration in Guangzhou than in other two cities. Chongqing which has high consumption of coal was found to have the highest mass concentration of Sulphur. Study on source apportionment and implication of pollutants in Chengdu by Tao et al. [17] found WSII accounting 42% of mass concentration of PM_2.5_. The dominant ions were ${\mathrm{SO}}_{4}^{2-}$, $\;{\mathrm{NO}}_{3}^{-}$ and ${\mathrm{NH}}_{4}^{+}$ which accounted 90% of the total ion concentration.

Wuhan, which is the capital city of Hubei Province (Longitude 113°41′~115°05′ E and Latitude 29°58′~31°22′ N) had a population of 10.91 million people in 2017. A number of studies [19,20,21,22,23,24,25,26] reported that this city experienced serious PM_2.5_ pollution events in recent years. Nevertheless, little has been done to study WSII which are the main constituents of PM_2.5_. Apart from the scanty literature on seasonal variation [14,15], nothing else can be found with regard to WSII in Wuhan. Therefore, this study is aimed at bridging the knowledge gap by analysing the composition of WSII in PM_2.5_, deposition flux and atmospheric boundary layer structure (ABLS). Note that, PM_2.5_ has been associated with negative health effects by a number of authors [27,28,29], so it worth to note its constituents as the first step to control it. Generally, we expect to provide results that will be useful in determining the important contributing sources of PM_2.5_ pollution which can be used in making policies in emission control. The results can also be used to validate models on removal mechanism and it can also act as the base of estimating the impact of particulate matter on the environment.

## 2. Methodology

### 2.1. Data and Sampling Site

The samples used in this study were collected from a roof top of the Institute of Atmospheric Physics and Atmospheric Environment, China University of Geosciences (Wuhan) at about 8 m from the ground (Longitude 114°23′ E and Latitude 30°31′ N). The sampling point is of interest as it is densely populated area with low traffic and there is no stationary emission sources near the area. A medium flow sampler (Type TH-150F, Wuhan Tianhong Company, Wuhan, China) was used for sampling daily concentration of PM_2.5_ using Quartz Fiber Filter membrane (QFF, Φ90 mm, Whatman Company, Maidstone, UK). A total number of 31 samples were collected from 8 January to 10 February 2015. The wet deposition samples were collected using two glass beaker devices with acapacity of 1000 mL every day at 0800 local standard time (LST) in strict accordance with the China national standards for the collection and preservation of samples [30]. Before the first use of the beaker, it was soaked with 10% (*v*/*v*) of nitric acid for the whole night and then washed with purified water and cleaned several times with deionized water. Thereafter, ion chromatography was used to determine if ${\mathrm{Cl}}^{-}$ concentration of deionized water from the beaker was the same as its counterpart before it was approved for sample collection. The beaker was then closed all over the time, and opened only when there was precipitation to avoid dry collection. After every use, it was washed with deionized water, dried and covered waiting for another use.

During the sampling period, a total number of five valid precipitation samples were collected. Dry deposition samples were also collected in accordance with the China national standard which recognizes a glass cylinder with diameter of 150 mm and height of 300 mm as a sampling instrument; the samples were then added to 50 mL of distilled water. Samples were collected after every five days at 0900 LST, and whenever precipitation occurred, the sampling process was stopped and started afresh. During the sampling process, two sets of experiments were set at each time, and the mean values of the two groups yielded a total number of seven valid samples.

### 2.2. Sample Analysis

Before sampling, the quartz membrane was placed in a muffle furnace at 450 °C for 5 h to remove possible substances that may exist. Before and after sampling, the membrane was packed in aluminium foil and kept at constant temperature (25 °C) and humidity (52%) for 24 h. The weight of the sample was measured by using an electronic analytical balance (T-114 type, Sartorius Company, Göttingen, Germany) which was then used to calculate the PM_2.5_ concentration. Thereafter, a half quartz membrane punch was soaked in 50 mL of polypropylene centrifuge tube, and 15 mL of ultrapure water was added, before it was placed under ultrasonic extraction for 30 min. The extraction temperature was set at 20 °C~30 °C through a micro porous filter membrane of 0.45 µm. The same procedure was done during the treatment of blank quartz membrane and repeated two times. Dry and wet deposition samples were immediately packed into two small colourless polyethylene plastic bottles after collection and kept refrigerated at 4 °C to avoid contamination before laboratory analysis. 

The anion (${\mathrm{Cl}}^{-}$, ${\mathrm{NO}}_{3}^{-}$ and ${\mathrm{SO}}_{4}^{2-}$) concentration was measured using an ion chromatograph (ICS-1100) whilst the cations concentration ($K^{+}$, ${\mathrm{Ca}}^{2+}$, ${\mathrm{Na}}^{+}$, ${\mathrm{Mg}}^{2+}$ and ${\mathrm{NH}}_{4}^{+}$) was measured using an inductively coupled plasma optical atomic emission spectrometer (ICAP6300, Thermo Fisher Scientific Inc., Waltham, MA, USA) after 24 h of refrigeration. Mean and standard deviation of anions and cations were calculated to determine their variation tend in each sample. Moreover, the correlation analysis was defined as significant if the probability (*p*-value) of the test was less than 0.05. The concentration of ions, mass concentrations in PM_2.5_, and deposition flux were calculated using Equations (1)–(3), respectively:

(1) $$\mathrm{Ions}\;\mathrm{concentration}\;\mathrm{in}\;{\mathrm{PM}}_{2.5}\;=\;C\times V_{1}\times 4\times 1000/V_{2}$$

where:

C: Determined ion concentration (mg/L) in volume of a sample
V_1_: Volume (L) after dilution of the sample solution taken at the time of measurement
V_2_: Sample volume of air in standard state (m^3^)

(2) $${\mathrm{PM}}_{2.5}\;\mathrm{mass}\;\mathrm{concentration}\;=\;(M_{2}-M_{1})\times 10^{6}/V_{2}$$

where:

M_1_: Mass (g) of the filter before sampling
M_2_: Mass (g) of the filter after sampling

Atmospheric dry and wet deposition flux (mg·m^−2^·month^−1^)

F = Q_t_/S (3)

where:

F: Deposition flux (mg·m^−2^·month^−1^)
Q_t_: Total settlement (mg·month^−1^)
S: Sampling area (m^2^)
S = 3.14 × D^2^/4/10^4^
D: Diameter of the sampler (cm)

Studies by Shen [1] and Kulshrestha [7] used a neutralizing factor (NF) to describe the interaction between anions and cations. This study also calculated NF of dominant cations; ${\mathrm{Ca}}^{2+}$, ${\mathrm{NH}}_{4}^{+}$ and ${\mathrm{Mg}}^{2+}$ using their equivalent concentrations by the following equations:

(4) $${\mathrm{NF}}_{{\mathrm{Ca}}^{2+}}\;=\;\frac{{\mathrm{Ca}}^{2+}}{{\mathrm{SO}}_{4}^{2-}+{\mathrm{NO}}_{3}^{-}}$$

(5) $${\mathrm{NF}}_{{\mathrm{NH}}_{4}^{+}}\;=\;\frac{{\mathrm{NH}}_{4}^{+}}{{\mathrm{SO}}_{4}^{2-}+{\mathrm{NO}}_{3}^{-}}$$

(6) $${\mathrm{NF}}_{{\mathrm{Mg}}^{2+}}\;=\;\frac{{\mathrm{Mg}}^{2+}}{{\mathrm{SO}}_{4}^{2-}+{\mathrm{NO}}_{3}^{-}}$$

### 2.3. HYSPLIT Model and Meteorological Data

The Hybrid Single-Particle Lagrangian Integrated Trajectory (HYSPLIT) model developed by the U.S. National Oceanic and Atmospheric Administration (NOAA) Air Resources Laboratory (ARL) was used to track air mass trajectories [31,32]. It has a relatively complete transport, diffusion and sedimentation model for handling a variety of meteorological element input fields, multiple physical processes and different types of pollutant emission sources (http://www.arl.noaa.gov/HYSPLIT.php). HYSPLIT model was used to track back air trajectories of Wuhan for 72 h, with a view to the qualitative description of the air transport path. Meteorological data input was from National Centers for Environmental Prediction (NCEP) fields obtained from NOAA, which are available at every 3 h with 1° × 1° spatial resolution. Three different levels (100 m, 300 m and 600 m) were set within the model to determine the specific transport path. This study also used L-band radar sounding data and ground meteorological variables provided by the Wuhan Meteorological Bureau. The daily observation time for L-band radar was 0700 LST at a vertical resolution of 10 m. Vertical profile of different meteorological variables (temperature, relative humidity, wind speed and direction) from the ground up to 3000 m was then used to describe the atmospheric boundary layer structure (ABLS). L-band sound data were used to plot the vertical profile of the meteorological variables. The characteristics of ABLS and the pollutants were further studied during clean and high pollution days.

## 3. Results and Discussion

### 3.1. Analysis of PM_2.5_ Concentration

**Figure 1:**
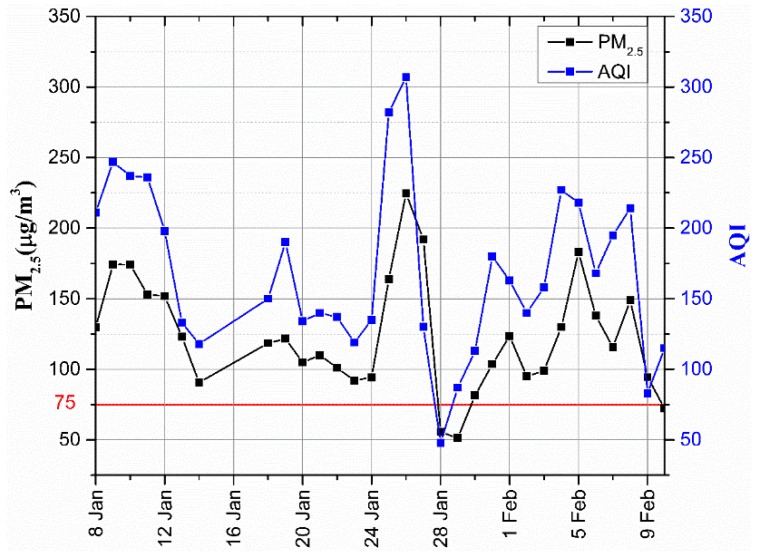
Daily average PM_2.5_ concentration. AQI: Air Quality Index.

**Figure 2:**
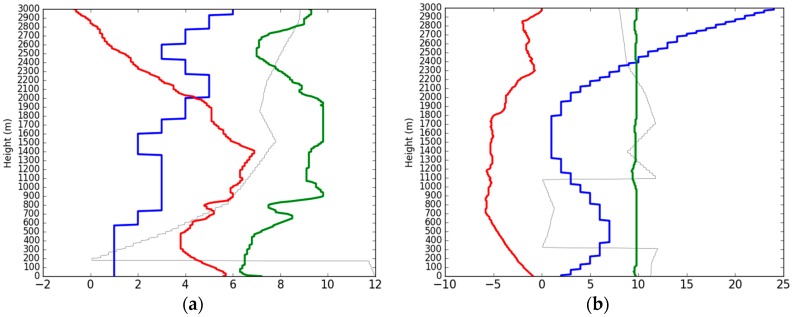
Vertical structure of the atmosphere at 0700 LST on (**a**) Highest concentration of PM_2.5_ (**b**) Lowest concentration of PM_2.5_. The line shows the profile of temperature (red, °C), relative humidity (green, %), wind velocity (blue, m/s), and dominant wind direction (grey). The number on the *x*-axis is the result after dividing the relative humidity value by 10 and wind direction angle by 30 while temperature and wind velocity remain the same, the *y*-axis is height (m).

During the sampling period, daily concentration of PM_2.5_ at the sampling site was higher and above the daily set limit of 75 µg/m^3^ [33] by a frequency of 94%. Moreover, during these days, there was no single day which met the United State Environmental Protection Agency (US EPA) and World Health Organisation (WHO) daily set PM_2.5_ limits (35 µg/m^3^ and 25 µg/m^3^, respectively) [34]. As it can be seen in Figure 1, the highest and lowest concentration of PM_2.5_ were on 26 and 29 January, corresponding to 224.64 µg/m^3^ and 51.32 µg/m^3^, respectively. The AQI during these days was 307 and 87, indicating the air quality was “severe pollution” and “good”, respectively (Table 1). For the whole period of 31 days, only three days; 28, 29 January, and 10 March, were within the set daily limit, and the AQI during these days was 48, 87, and 115 corresponding to “excellent”, “good” and “slightly polluted” air quality, respectively. Similar results were reported by nine air quality monitoring stations for the whole city managed by Wuhan Environment Protection Bureau (WHEPB) (http://hbj.wuhan.gov.cn/airInfoView.jspx). It should be noted that, these nine stations are distributed all over Wuhan city, and are the ones used to calculate the AQI of the city. The average concentration of PM_2.5_ from these stations show that the highest concentration (253.71 µg/m^3^) and the lowest concentration (32.82 µg/m^3^) were on the same date, that is on 26 and 29 January, respectively. Moreover, the mean and standard deviations of PM_2.5_ at the sampling site were 122.95 µg/m^3^ and 40.30 µg/m^3^ while that of the whole city were 127.49 µg/m^3^ and 48.99 µg/m^3^, respectively. Based on the above analysis, it was clear that the sampling site and Wuhan city were polluted during the sampling period mainly by the PM_2.5_. This was not a surprise, as a number of previous studies have reported Wuhan to be polluted by PM_2.5_ during winter [14,20,25]. Analysis of the ABLS during these two days show that, high concentration of PM_2.5_ was accompanied by dry and warm ABLS, weak low level wind and strong inversion layer (Figure 2a). An opposite scenario was observed when the PM_2.5_ concentration was low, as the whole ABLS was cool and wet, with higher low level wind and the disappearence of the inversion layer (Figure 2b). These general characteristics during high (low) concentration of PM_2.5_ are unfavourable (favourable) for dispersion and mixing of aerosols. Corroborated results to this have been reported in our previous works [21,26].

### 3.2. Concentration of Water-Soluble Inorganic Ions

Analysis of PM_2.5_ showed that the concentration of WSII accounted for 42% of the total concentration. This was not a surprise as it was also reported by a number of authors elsewhere [2,13,16,17], that WSII occupy a significant portion of PM_2.5_ concentration. Among these WSII compounds, ${\mathrm{NO}}_{3}^{-}$ occupied the major portion (42%), followed by ${\mathrm{SO}}_{4}^{2-}$ (33%) and ${\mathrm{NH}}_{4}^{+}$ (12%), while ${\mathrm{Mg}}^{2+}$, ${\mathrm{Na}}^{+}$ and $K^{+}$ occupied about 4%. Higher concentration of ${\mathrm{NO}}_{3}^{-}$ and ${\mathrm{SO}}_{4}^{2-}$ in the PM_2.5_ sample suggests significant contribution of mobile and stationary source pollutants to air pollution in Wuhan city. Concentration range of ${\mathrm{NO}}_{3}^{-}$ was 2.05–46.20 µg/m^3^, ${\mathrm{SO}}_{4}^{2-}$ was 6.78–38.90 µg/m^3^, ${\mathrm{NH}}_{4}^{+}$ was 2.71–20.71 µg/m^3^, ${\mathrm{Ca}}^{2+}$ was 0.16–8.82 µg/m^3^, ${\mathrm{Cl}}^{-}$ was 0.40–5.53 µg/m^3^, $K^{+}$ was 0.21–2.28 µg/m^3^, ${\mathrm{Na}}^{+}$ was 0.04–2.34 µg/m^3^, and ${\mathrm{Mg}}^{2+}$ was 0.01–0.26 µg/m^3^. Similar variation range of these WSII have been reported by Ianniello et al. [35]. Moreover, it was further found that the correlation coefficient (*p*-value) between mass concentration of PM_2.5_ and the sum of all WSII was 0.50 (0.0019), while the correlation between PM_2.5_ and the sum of three inorganic ions which occupied the highest portion (${\mathrm{NO}}_{3}^{-}$, ${\mathrm{SO}}_{4}^{2-}$ and ${\mathrm{NH}}_{4}^{+}$) was 0.49 (0.0026). This indicates that these three ions are the main determinants of the PM_2.5_ concentration. Table 2 shows the correlation coefficient between individual WSII, high correlation is between ${\mathrm{NH}}_{4}^{+}$ and ${\mathrm{Cl}}^{-}$ (r = 0.76 and *p*-value = 0.0000004) and followed by the one between ${\mathrm{NH}}_{4}^{+}$ and ${\mathrm{NO}}_{3}^{-}$ (r = 0.71 and *p*-value = 0.0000046), since they can exist as ammonium salt in atmospheric aerosols (i.e., ${\mathrm{NH}}_{4}\mathrm{Cl}$ and ${\mathrm{NH}}_{4}{\mathrm{NO}}_{3}$). Likewise, high correlation coefficient was between ${\mathrm{NO}}_{3}^{-}$ and ${\mathrm{SO}}_{4}^{2-}$ (r = 0.71 and *p*-value = 0.0000036), because they can originate from the same source. 

#### 3.2.1. Equivalent Ratio of ${\mathrm{NO}}_{3}^{-}$/${\mathrm{SO}}_{4}^{2-}$ in PM_2.5_

**Figure 3:**
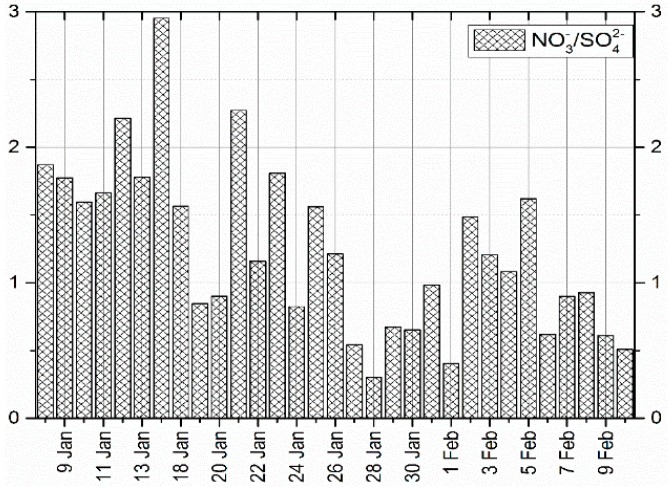
Equivalent ratio of ${\mathrm{NO}}_{3}^{-}$/${\mathrm{SO}}_{4}^{2-}$ in PM_2.5._

**Figure 4:**
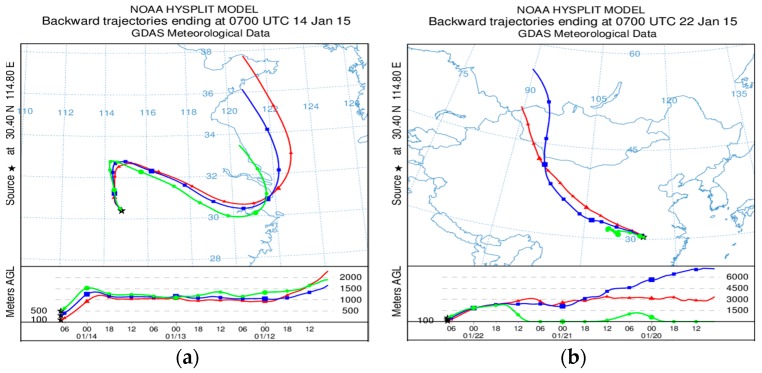
A 72 h back trajectory results when (**a**) Equivalent ratio was greater than 1 (**b**) Equivalent ratio was less than 1.

During the sampling process the equivalent ratios of ${\mathrm{NO}}_{3}^{-}$/${\mathrm{SO}}_{4}^{2-}$ in PM_2.5_ of greater than 1 was 55% and the equivalent ratio of less than one (1) was 45%. The observed higher ratio of nitrogen may be contributed partially by traffic on Lu Mo road which is about 500 m from the sampling site while sulphur may originate from a coal fired power plant (Yangluo Power Station) which is located in the North North East (NNE) of the city [19]. We note that this ratio has been widely used by a number of authors [16,36,37] to reflect the relative contribution of stationary emission sources (e.g., coal combustion) and mobile emissions (e.g., motor vehicles and ship emission) of nitrogen and sulphur in the atmosphere. When the equivalent ratio is greater than 1, it indicates that, most of the pollutants are derived from mobile sources, and when it is less than 1, then most of the pollutants are derived from stationary sources. For this case, there is no significant difference between mobile and stationary source emissions, therefore conclusion cannot be drawn at this stage. However, Figure 3 shows that, before 18 January, the pollutants were mainly from mobile sources, while in between 27 January and 1 February, and after 5 February, the pollutants were mainly from stationary sources. Moreover, the maximum and minimum equivalent ratios were 2.95 and 0.30 on 14 and 28 January, respectively. It is worth noting that these ratios can be affected by trans-boundary movement of air pollutants. In order to analyze the effect of trans-boundary movement of pollutants, HYSPLIT model was used to track back the upper air mass of Wuhan city when the equivalent ratio was the highest and lowest. Figure 4a shows the 72 h back trajectories result of air mass which arrived in Wuhan when the equivalent ratio was the highest. The air mass originated from the Yellow Sea, the area which is clean but it passed through Shanghai, Zhejiang and Anhui, the areas which face a serious pollution mainly from mobile sources [38]. This might be one of the reasons why during this day the pollutants from the mobile sources exceeded those from stationary sources, because the air in Wuhan was fed by more pollutants of nitrogen origin. On the other hand, Figure 4b shows that when the equivalent ratio was below 1, the air masses originated from Gansu Province and crossed Sichuan and Chongqing before they arrived in Wuhan. The source and route of this air mass is famous in the production and usage of coal, thus, it is widely affected by pollutants of sulphur origin [16,39]. This observed air mass are thought to enrich Wuhan with pollutants of sulphur origin which ended up lowering the equivalent ratio.

#### 3.2.2. Analysis of Water-Soluble Inorganic Ions in Wet Deposition

A total of five wet deposition samples were collected during the sampling period while chemical composition of eight (8) WSII (${\mathrm{Na}}^{+}$, $K^{+}$, ${\mathrm{Ca}}^{2+}$, ${\mathrm{Mg}}^{2+}$, ${\mathrm{NH}}_{4}^{+}$, ${\mathrm{SO}}_{4}^{2-}$, ${\mathrm{NO}}_{3}^{-}$ and ${\mathrm{Cl}}^{-}$) were studied. As it can be seen in Table 3, the concentration of ions were higher on 26 January, consistent with the PM_2.5_ concentration, while on 1 February, $K^{+}$ was not detected and the concentration of almost all other WSII where very low on this day and a day after (2nd of February). One of the reasons for the low concentrations of ions recorded in these two days may be the rainfall which occurred at the end of January and thus reduced the concentration of PM_2.5_ in the atmosphere. This observed result is concurrent with the PM_2.5_ mass concentration and AQI recorded during these days (Figure 1) as the days with lower concentration of PM_2.5_ coincided with rainfall days. Corroborated results to this has been reported by Gambaro [8] and Pan [40] due to scavenging effect precipitation. A closer look at the concentration of each ion show that ${\mathrm{SO}}_{4}^{2-}$ was the highest in concentration occupying 39%, followed by ${\mathrm{NO}}_{3}^{-}$ which occupied 31%. Other ions; ${\mathrm{Cl}}^{-}$ occupied 12%, ${\mathrm{Ca}}^{2+}$ occupied 7%, ${\mathrm{NH}}_{4}^{+}$ occupied 5%, ${\mathrm{Na}}^{+}$ occupied 3%, $K^{+}$ occupied 2% and ${\mathrm{Mg}}^{2+}$ occupied 1%. Moreover, average concentration of ${\mathrm{SO}}_{4}^{2-}$ and ${\mathrm{Cl}}^{-}$ were 12.305 mg/L and 3.981 mg/L, respectively, far below the set limit of 250 mg/L by the China environmental quality standard for surface water for both ions [41]. Therefore, this indicates that the ions can contaminate surface water but cannot cause water pollution. Furthermore, the correlation coefficient between ${\mathrm{SO}}_{4}^{2-}$ and ${\mathrm{NO}}_{3}^{-}$ was the best (r = 0.99 and *p*-value = 0.0007), followed by ${\mathrm{Mg}}^{2+}$ and ${\mathrm{Ca}}^{2+}$ (r = 0.94 and *p*-value = 0.0088) and ${\mathrm{NO}}_{3}^{-}$ and ${\mathrm{Cl}}^{-}$ (r = 0.93 and *p*-value = 0.0106), because they are from the same source. The correlation coefficient between ${\mathrm{NH}}_{4}^{+}$ and ${\mathrm{SO}}_{4}^{2-}$ was also excellent (r = 0.91 and *p*-value = 0.0153) since these ions can exist as ammonium salt $({\left({\mathrm{NH}}_{4}\right)}_{2}{\mathrm{SO}}_{4}$) in atmospheric aerosol.

**Figure 5:**
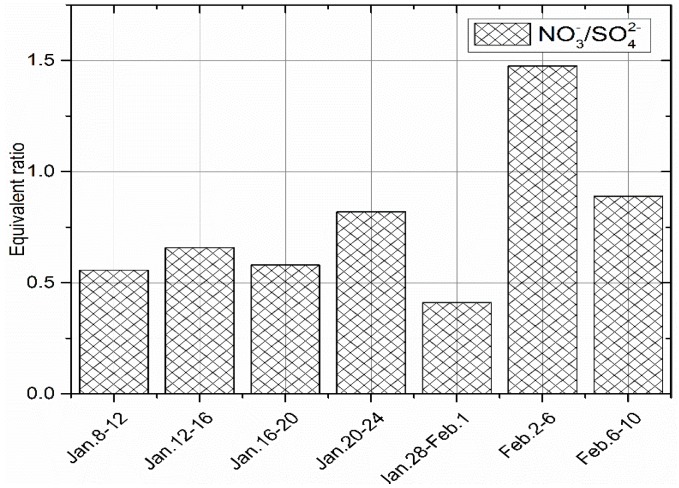
Equivalent ratio of ${\mathrm{NO}}_{3}^{-}$/${\mathrm{SO}}_{4}^{2-}$ on dry deposition.

Interestingly, Figure 5 shows the equivalent ratio of ${\mathrm{NO}}_{3}^{-}$/${\mathrm{SO}}_{4}^{2-}$ during wet depositions to be below 1 on all the days, which suggests that the stationary source (coal combustion) was the main source of air pollutants. The maximum and minimum equivalent ratio were 0.98 and 0.57 on 24 January and 2 February, respectively. Furthermore, Table 4 shows the removal efficiency of precipitation on PM_2.5_ and WSII. The first batch of rainfall was found to be excellent in removing $K^{+}$, followed by ${\mathrm{NO}}_{3}^{-}$, ${\mathrm{Na}}^{+}$ and ${\mathrm{Mg}}^{2+}$, while the removal efficiency of the rest were below 50%. Corroborated result to this have been reported by Saxena et al. [12]. An opposite scenario happened in the case of ${\mathrm{Cl}}^{-}$, as it exhibited an increasing trend, suggesting that the pollution source discharged more air pollutants containing ${\mathrm{Cl}}^{-}$ to the atmosphere during the rainfall period than before. The second batch of rainfall show that it was excellent in removing ${\mathrm{Cl}}^{-}$ and good in removing ${\mathrm{NO}}_{3}^{-}$. Moreover, its efficiency in removing $K^{+}$, ${\mathrm{Ca}}^{2+}$, ${\mathrm{NH}}_{4}^{+}$ and PM_2.5_ was below 50%.

#### 3.2.3. Analysis of Water-Soluble Inorganic Ions in Dry Deposition

**Figure 6:**
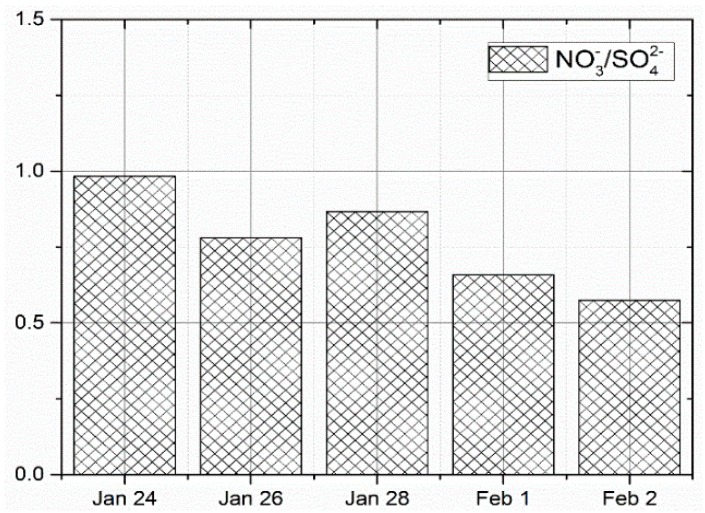
Equivalent ratio of ${\mathrm{NO}}_{3}^{-}$/${\mathrm{SO}}_{4}^{2-}$ on wet deposition.

During the sampling period, dry deposition samples were also collected by wet deposition method. A total of seven dry deposition samples were collected and WSII were analyzed. As it can be seen in Table 5, the order of mass concentration of WSII on dry deposition was ${\mathrm{SO}}_{4}^{2-}$ > ${\mathrm{NO}}_{3}^{-}$ > ${\mathrm{Cl}}^{-}$ > ${\mathrm{Ca}}^{2+}$ > ${\mathrm{NH}}_{4}^{+}$ > ${\mathrm{Na}}^{+}$> $K^{+}$ > ${\mathrm{Mg}}^{2+}$, which is consistent with what was observed on wet deposition. Saxena et al. [12] reported similar results to this and linked the dry deposition of ${\mathrm{Ca}}^{2+}$, ${\mathrm{Na}}^{+}$, $K^{+}$ and ${\mathrm{Mg}}^{2+}$ with soil derived aerosols carried into the atmosphere by the action of wind. As it was the case for wet deposition, the average concentration of ${\mathrm{SO}}_{4}^{2-}$ and ${\mathrm{Cl}}^{-}$ were 6.37 mg/L and 3.41 mg/L, respectively, which are far below the national set standard for surface water of 250 mg/L [41]. Likewise, the correlation coefficient results show that there was a good correlation between anions and cations. For instance, the correlation coefficient between ${\mathrm{Ca}}^{2+}$ and ${\mathrm{Mg}}^{2+}$ was found to be 0.84 with the *p*-value of 0.0027, was the best, followed by ${\mathrm{SO}}_{4}^{2-}$ and ${\mathrm{Cl}}^{-}$ (r = 0.76 and *p*-value = 0.0116), because they are from the same source. Furthermore, the correlation coefficient between ${\mathrm{SO}}_{4}^{2-}$ and ${\mathrm{Mg}}^{2+}$ was found to be 0.76 and their *p*-value was 0.0126, because they can exist as ${\mathrm{MgSO}}_{4}$ in atmospheric aerosol. Additionally, the equivalent ratio of ${\mathrm{NO}}_{3}^{-}$/${\mathrm{SO}}_{4}^{2-}$ on dry deposition was less than 1 in almost all the samples, except the one of 2–6 February (Figure 5). These results are consistent with the results obtained from wet deposition samples (Figure 6) and PM_2.5_ concentration ahead of 28 January (Figure 3). Therefore, this suggests that the source of air pollutants during the sampling campaign was mainly from a stationary source.

### 3.3. Estimation of Deposition Flux of Water-Soluble Inorganic ions

The total dry and wet depositions flux of WSII during the sampling period was 5834.42 mg·m^−2^·month^−1^. The deposition flux of ${\mathrm{SO}}_{4}^{2-}$ was the highest for anions reaching 1912.17 mg·m^−2^·month^−1^, while the deposition flux of ${\mathrm{Ca}}^{2+}$ was the highest for cations reaching 585.28 mg·m^−2^·month^−1^. The order of deposition flux was ${\mathrm{SO}}_{4}^{2-}$ > ${\mathrm{NO}}_{3}^{-}$ > ${\mathrm{Cl}}^{-}$ > ${\mathrm{Ca}}^{2+}$ > ${\mathrm{NH}}_{4}^{+}$ > ${\mathrm{Na}}^{+}$> $K^{+}$ > ${\mathrm{Mg}}^{2+}$, as it was the case for dry deposition (Table 6). Moreover, the highest deposition flux of anions for dry and wet deposition was the one of ${\mathrm{SO}}_{4}^{2-}$, equivalent to 841.55 mg·m^−2^·month^−1^ and 1070.62 mg·m^−2^·month^−1^, respectively. For the case of cations, ${\mathrm{Ca}}^{2+}$ was the highest in dry deposition contributing 388.44 mg·m^−2^·month^−1^, while ${\mathrm{Na}}^{+}$ was the highest in wet deposition contributing 246.02 mg·m^−2^·month^−1^. Additionally, Table 7 show the ionic balance (∑cations/∑anions) in wet deposition samples are very different from each other. The highest anions were those of ${\mathrm{SO}}_{4}^{2-}$ and ${\mathrm{NO}}_{3}^{-}$ with the total concentration of 1.29 meq/L and 0.82 meq/L while the highest cations were those of ${\mathrm{Ca}}^{2+}$ and ${\mathrm{NH}}_{4}^{+}$ with the total concentration of 0.57 meq/L and 0.42 meq/L, respectively. The lowest anions and cations in concentration were ${\mathrm{Cl}}^{-}$ and $K^{+}$ with the concentration of 0.56 meq/L and 0.08 meq/L, respectively. This shows that the ions in the wet deposition sample are not balanced, and the cations are much lower than the anions. The following can be the reasons for this: (1) important cations were not included in the analysis (2) some rare cations are too high in concentration. Furthermore, the concentration of ${\mathrm{SO}}_{4}^{2-}$ and ${\mathrm{NO}}_{3}^{-}$ accounted for 48% and 30% of anions, respectively, and their ratio (${\mathrm{SO}}_{4}^{2-}$/${\mathrm{NO}}_{3}^{-}$) was 1.59, indicating that the acidity of precipitation is mainly from Sulphate. The average NF for ${\mathrm{Ca}}^{2+}$, ${\mathrm{NH}}_{4}^{+}$ and ${\mathrm{Na}}^{+}$ in wet deposition were 0.27, 0.20 and 0.08, indicating that, ${\mathrm{Ca}}^{2+}$ and ${\mathrm{NH}}_{4}^{+}$ were the main neutralizers of precipitation. Similar observation to this has been reported in study by Shen [1] in northwest China. These cations; ${\mathrm{Ca}}^{2+}$ and ${\mathrm{NH}}_{4}^{+}$ accounted for 42% and 30%, respectively, while their ratio (${\mathrm{Ca}}^{2+}$/${\mathrm{NH}}_{4}^{+}$) was 1.37, suggesting the alkaline substance in precipitation was mainly dominated by Calcium salts. Therefore, this implies that the main pollutants which can affect surface water in wet deposition are mainly from Sulphate and Calcium salt. Table 8 shows the ratio of anions and cations in a dry deposition sample during the sampling campaign. Basically, the amount of anions and cations in the dry deposition sample are balanced, and the sum of the cations are higher than the sum of the anions. Similar to wet deposition, anions with the highest concentration were ${\mathrm{SO}}_{4}^{2-}$ (49.96 mg/L) and ${\mathrm{NO}}_{3}^{-}$ (40.56 mg/L) and the cations were ${\mathrm{Ca}}^{2+}$ (23.06 mg/L) and ${\mathrm{NH}}_{4}^{+}$ (17.41 mg/L). The concentration of ${\mathrm{SO}}_{4}^{2-}$ and ${\mathrm{Cl}}^{-}$ in dry deposition sample accounted for 43% and 31%, respectively of the anions, and their ratio (${\mathrm{SO}}_{4}^{2-}$/${\mathrm{Cl}}^{-}$) was 1.4, indicating the acidity in dry deposition was mainly from sulphate. The NF for ${\mathrm{Ca}}^{2+}$, ${\mathrm{NH}}_{4}^{+}$ and ${\mathrm{Na}}^{+}$ were 0.25, 0.19 and 0.11, respectively, suggesting that, ${\mathrm{Ca}}^{2+}$ and ${\mathrm{NH}}_{4}^{+}$ were the main neutralizers as it was the case for wet deposition. Concentration of ${\mathrm{Ca}}^{2+}$ and ${\mathrm{NH}}_{4}^{+}$ occupied 44% and 32%, respectively of the ions and their ratio (${\mathrm{Ca}}^{2+}$/${\mathrm{NH}}_{4}^{+}$) was 1.36, indicating that the alkaline substance was mainly from calcium salts. Therefore, sulfate and calcium salts are the main pollutants of surface water in dry deposition, even though their concentration is much lower to cause pollution. Additionally, the total mass of PM_2.5_ collected during the sampling campaign was 460.35 mg, while the total mass of eight (8) water soluble ions was 94.48 mg, equivalent to 21% of the total mass. That is to say, about 21% of the PM_2.5_ are deposited on the earth’s surface and surface water resources can end up affecting the quality of water even though it cannot cause pollution.

## 4. Conclusions

This study measured PM_2.5_ concentration, dry and wet depositions, and deposition flux of WSII at the sampling site located in China University of Geosciences (Wuhan). The average concentration of PM_2.5_ at the sampling site during the whole sampling period of 31 days was 122.95 µg/m^3^, while the average concentration of PM_2.5_ for the whole city was 127.49 µg/m^3^. For the whole sampling period, PM_2.5_ concentration was above the National set limit by 94%, and above US EPA and WHO daily set limits in all the sampling days. The concentration of WSII in PM_2.5_ occupied 42%, whereby ${\mathrm{NO}}_{3}^{-}$, ${\mathrm{SO}}_{4}^{2-}$, and ${\mathrm{NH}}_{4}^{+}$ were the dominant ions in the sample as they contributed 87% of the total concentration of WSII. Three kinds of ions (${\mathrm{SO}}_{4}^{2-}$, ${\mathrm{NO}}_{3}^{-}$ and ${\mathrm{Cl}}^{-}$) were the main ions in the precipitation samples as they accounted 82% of the total concentration. The ions concentration of ${\mathrm{SO}}_{4}^{2-}$ and ${\mathrm{NO}}_{3}^{-}$ were the highest in the PM_2.5_ sample, dry and wet depositions. The lowest ions concentration were those of ${\mathrm{Na}}^{+}$, $K^{+}$ and ${\mathrm{Mg}}^{2+}$ for PM_2.5_ sample, dry and wet deposition. Basically, the lowest and highest concentration of ions were the same in all the samples during this study. Furthermore, the highest correlation coefficient (r) between the WSII was found in wet deposition sample (${\mathrm{SO}}_{4}^{2-}$ and ${\mathrm{NO}}_{3}^{-}$) of 0.99, followed by the one of dry deposition (${\mathrm{Ca}}^{2+}$ and ${\mathrm{Mg}}^{2+}$) of 0.84 and in PM_2.5_ sample (${\mathrm{NH}}_{4}^{+}$ and ${\mathrm{Cl}}^{-}$) was 0.76. The acidity and alkalinity of both dry and wet deposition samples were mainly from sulphate and calcium, respectively. The NF calculation indicate ${\mathrm{Ca}}^{2+}$ and ${\mathrm{NH}}_{4}^{+}$ were the main neutralizers for both dry and wet deposition. These results indicate that, the main contaminants of surface water are principally sulfate and calcium salts, even though their concentrations are much lower to cause pollution. The average removal efficiency of ${\mathrm{Na}}^{+}$, ${\mathrm{Mg}}^{2+}$ and ${\mathrm{NO}}_{3}^{-}$ was above 66%, while the removal efficiency of PM_2.5_ and the remaining WSII was below 55%. Also, the deposited sample of WSII can contaminate surface water, even though they may not cause pollution.

## Figures and Tables

**Table 1:** Air quality grading standards.

Air Quality Index (AQI)	Air Quality Index Level	Air Quality Index Category
0–50	Level 1	Excellent
51–100	Level 2	Good
101–150	Level 3	Slightly polluted
151–200	Level 4	Moderate polluted
201–300	Level 5	Heavy pollution
>300	Level 6	Severe pollution

**Table 2:** Correlation coefficient between PM_2.5_ and water-soluble inorganic ions.

Components	PM_2.5_	Cl^−^	NO_3_^−^	SO_4_^2−^	Ca^2+^	Mg^2+^	K^+^	Na^+^	NH_4_^+^
**PM_2.5_**	1	0.33	0.47	0.39	0.26	0.30	0.18	0.04	0.40
**Cl^−^**		1	0.69	0.49	0.04	0.03	0.51	0.12	0.76
**NO_3_^−^**			1	0.71	0.21	0.05	0.53	0.09	0.71
**SO_4_^2−^**				1	0.08	0.10	0.26	0.13	0.40
**Ca^2+^**					1	0.22	0.11	0.21	0.17
**Mg^2+^**						1	0.18	0.14	0.07
**K^+^**							1	0.18	0.55
**Na^+^**								1	0.37
**NH_4_^+^**									1

**Table 3:** Concentration of water-soluble inorganic ions in wet deposition.

Precipitation	Cl^−^	NO^3−^	SO_4_^2−^	Ca^2+^	Mg^2+^	K^+^	Na^+^	NH_4_^+^
	mg/L	mg/L	mg/L	mg/L	mg/L	mg/L	mg/L	mg/L
24 January	4.07	10.21	10.39	3.69	0.63	1.13	1.21	0.81
26 January	4.75	22.16	28.41	2.30	0.32	1.05	0.74	3.29
28 January	4.57	12.22	14.10	0.99	0.9	0.02	0.49	1.63
1 February	3.22	3.00	4.56	0.79	0.04	BDL	0.74	1.39
2 February	3.32	2.34	4.07	3.54	0.38	0.36	1.10	0.30

“BDL” means Below Detectable Limit.

**Table 4:** Concentration of PM_2.5_ (mg/L) before and after rainfall.

Date	Cl^−^	NO_3_^−^	SO_4_^2−^	Ca^2+^	Mg^2+^	K^+^	Na^+^	NH_4_^+^	PM_2.5_ Concentration
23 January 2015–28 January 2015	0.8	23.44	12.96	6.66	0.16	1.66	0.77	3.63	91.95
	2.08	5.45	10.05	5.36	0.05	0.21	0.18	3.10	55.61
Difference	−1.28	17.99	2.91	1.30	0.11	1.45	0.59	0.53	36.34
Removal efficiency	-	77%	22%	19%	70%	87%	77%	14%	40%
31 January 2015–2 February 2015	2.37	17.58	17.89	0.73	0.05	0.83	0.29	4.90	103.67
	0.45	7.57	18.74	0.72	0.05	0.67	0.45	3.94	95.04
Difference	1.92	10.01	−0.85	0.01	0.00	0.16	−0.16	0.96	8.63
Removal efficiency	82%	57%	-	2%	-	18%	-	20%	8%

**Table 5:** Mean and standard deviation values for dry deposition.

Components	Cl^−^	NO_3_^−^	SO_4_^2−^	Ca^2+^	Mg^2+^	K^+^	Na^+^	NH_4_^+^
	mg/L	mg/L	mg/L	mg/L	mg/L	mg/L	mg/L	mg/L
Mean	3.41	5.14	6.37	3.10	0.23	0.34	1.36	2.05
Standard deviation	0.16	3.04	1.66	1.06	0.09	0.35	0.86	0.68

**Table 6:** Deposition flux of water-soluble inorganic ions during dry and wet deposition.

Monthly Deposition Flux (mg·m^−2^·month^−1^)							
Cl^−^	NO_3_^−^	SO_4_^2−^	Ca^2+^	Mg^2+^	K^+^	Na^+^	NH_4_^+^
804.17	1549.82	1912.17	585.28	55.03	84.81	422.20	422.93

**Table 7:** Concentration of anions and cations in wet deposition.

Date	Cl^−^	NO_3_^−^	SO_4_^2−^	Ca^2+^	Mg^2+^	K^+^	Na^+^	NH_4_^+^	Sum of Cations/Anions
	meq/L	meq/L	meq/L	meq/L	meq/L	meq/L	meq/L	meq/L	
24 January	0.12	0.17	0.22	0.18	0.05	0.03	0.05	0.05	0.71
26 January	0.13	0.36	0.59	0.12	0.03	0.03	0.03	0.18	0.36
28 January	0.13	0.20	0.29	0.05	0.01	0.01	0.02	0.09	0.29
1 February	0.09	0.05	0.10	0.04	0.01	-	0.03	0.08	0.67
2 February	0.09	0.04	0.09	0.18	0.03	0.01	0.05	0.02	1.32

**Table 8:** Concentration of anions and cations in dry deposition.

Date	Cl^−^	NO_3_^−^	SO_4_^2−^	Ca^2+^	Mg^2+^	K^+^	Na^+^	NH_4_^+^	Sum of Cations/Anions
	meq/L	meq/L	meq/L	meq/L	meq/L	meq/L	meq/L	meq/L	
8–12 January	0.09	0.04	0.10	0.15	0.02	0.01	0.04	0.07	1.26
12–16 January	0.10	0.07	0.14	0.16	0.02	0.01	0.03	0.15	1.19
16–20 January	0.09	0.06	0.14	0.22	0.02	0.03	0.05	0.15	1.62
20–24 January	0.09	0.08	0.18	0.16	0.01	0.01	0.13	0.06	1.05
28 January–1 February	0.09	0.03	0.09	0.08	0.01	0.01	0.03	0.12	1.19
2–6 February	0.10	0.17	0.15	0.10	0.01	0.00	0.04	0.11	0.62
6–10 February	0.10	0.13	0.19	0.22	0.03	0.01	0.08	0.14	1.14
